# Aβ re‐wires CSF1R‐induced MAPK pathway activation in MMC microglia

**DOI:** 10.1002/alz.092388

**Published:** 2025-01-03

**Authors:** Sara Bitarafan, Zachary C. Uhlig, Brendan R Tobin, Owen S Armentrout, Srikant Rangaraju, Levi B Wood

**Affiliations:** ^1^ Georgia Institute of Technology, Atlanta, GA USA; ^2^ Georgia institute of technology, Atlanta, GA USA; ^3^ Yale University School of Medicine, New Haven, CT USA

## Abstract

**Background:**

Mitogen activated protein kinase (MAPK) signaling is a critical regulator of microglial phenotype, including phagocytic function, cytokine expression, and motility, among others. Importantly, both canonical and non‐canonical MAPK signaling is directly activated by RTKs, including Interestingly, CSF1R, is activated by two agonists, CSF1 and IL‐34, which have been shown to activate the receptor in different ways that can lead to However, little is known about how the affect microglial MAPK signaling, and whether their effects are dependent on disease state/Aβ exposure. In this study, we hypothesized that IL‐34 and CSF‐1 elicit distinct patterns of MAPK signaling activation in microglia and MAPK activation would be dependent on whether the cells were exposed to Aβ.

**Method:**

We applied agonists of CSF1R (i.e., IL‐34, CSF1) to the mouse microglial cell (MMC) line, either pre‐treated with 500nM of Aβ1‐42 or vehicle (1% NH4OH) for 48hr. Cells were serum starved (1hr), stimulated with each agonist for 5min, then lysed for Luminex quantification of a panel of 10 MAPK phosphoproteins.

**Result:**

In the absence of Aβ1‐42, both CSF1R and IL‐34 stimulated both canonical (ERK) and non‐canonical (p38, JNK) MAPK signaling phospho‐proteins (Wilcox test, p<0.05 vs control). While Aβ1‐42 pre‐treatment resulted in diminished activation in canonical MAPK signaling (i.e., MEK, ERK) following either CSF1 or IL‐34 stimulation, CSF1 stimulation of non‐canonical MAPK signaling through p38 and JNK was preserved with Aβ1‐42 pre‐treatment. These findings indicate that CSF1‐induced signaling is robust regardless of Aβ1‐42 exposure. Furthermore, different agonists of the same receptor distinctly activate non‐canonical MAPK pathway signaling (p38). Collectively, this finding supports the overall hypothesis that CSF‐1R‐induced signaling is dependent on Aβ and Aβ pre‐treatments shift the canonical ERK signaling to non‐canonical p38 signaling.

**Conclusion:**

These findings show that different agonists of CSF1R can provoke distinct profiles of canonical and non‐canonical MAPK pathway signaling in the presence of Aβ1‐42. Understanding differences in agonist‐specific microglial signaling in healthy and diseased microglia will be essential to designing microglial‐targeted therapeutics for treatment of AD.

